# Molecular Epidemiology and Colistin Resistant Mechanism of *mcr*-Positive and *mcr*-Negative Clinical Isolated *Escherichia coli*

**DOI:** 10.3389/fmicb.2017.02262

**Published:** 2017-11-17

**Authors:** Qixia Luo, Wei Yu, Kai Zhou, Lihua Guo, Ping Shen, Haifeng Lu, Chen Huang, Hao Xu, Shaoyan Xu, Yonghong Xiao, Lanjuan Li

**Affiliations:** ^1^State Key Laboratory for Diagnosis and Treatment of Infectious Diseases, Collaborative Innovation Center for Diagnosis and Treatment of Infectious Diseases, First Affiliated Hospital of Medical School, College of Medicine, Zhejiang University, Hangzhou, China; ^2^Department of Infectious Diseases, Zhejiang Provincial People's Hospital, People's Hospital of Hangzhou Medical College, Hangzhou, China; ^3^Division of Hepatobiliary and Pancreatic Surgery, Department of Surgery, First Affiliated Hospital, School of Medicine, Zhejiang University, Hangzhou, China

**Keywords:** colistin, mcr-positive, mcr-negative, resistant mechanism, molecular epidemiology, clinical isolated *E. coli*

## Abstract

Transmissible colistin resistance mediated by the *mcr* gene has been reported worldwide, but clinical isolates of *mcr*-negative colistin-resistant *Escherichia coli* are rarely reported. The aim of this study was to evaluate the mechanism of colistin resistance among *mcr*-positive and *mcr*-negative *E. coli* clinical isolates by performing a molecular epidemiological surveillance. For the first time ever, we show nearly the same isolation ratio for *mcr*-negative and *mcr*-positive colistin-resistant clinical isolates (47.5 and 52.5%, respectively), with no demonstrable nosocomial transmission. We provide evidence for the prevalence of the *mcr*-positive IncX4 plasmid and its high potential for horizontal transfer, with no obvious sequence type (ST) preference. In addition, the minimal inhibitory concentrations (MICs) of colistin of the *mcr*-negative *E. coli* isolates were obviously higher than those of *mcr*-positive isolates. Apart from the usually detected genes, i.e., *pmrAB, phoPQ*, and *mgrB*, other genes may be associated with the colistin resistance in *mcr*-negative *E. coli*. To the best of our knowledge, this is the first paper to report the molecular epidemiological surveillance and the proper mechanism of colistin resistance in *mcr*-negative *E. coli* clinical isolates. Together, the results show that colistin resistance was prevalent not only in the *mcr*-positive clinical *E. coli* isolates but also in the *mcr*-negative isolates.

## Introduction

Polymyxin, recently reintroduced in human medicine practice, constitutes one of the last resorts for the treatment of multidrug-resistant Gram-negative bacteria (Nation et al., 2014). Colistin (polymyxin E), with pronounced antimicrobial activity against Gram-negative bacteria, is a cyclic polycationic peptide that interacts with anionic lipopolysaccharide (LPS) molecules (Olaitan et al., 2014). Even though LPS is the initial target, the exact mechanism underpinning the effect of polymyxins on Gram-negative bacteria remains unclear (Poirel et al., 2017).

A plasmid-mediated colistin resistance gene, *mcr-1*, harbored by *Escherichia coli* and *Klebsiella pneumoniae* isolated from animals and hospital inpatients, was first reported in China (Liu et al., 2016). Then it became popular all over the world (Rapoport et al., 2016; Rolain et al., 2016; Sonnevend et al., 2016), demonstrating a horizontal transfer mechanism for colistin resistance. Additional novel plasmid-encoded colistin resistance genes were identified as well: *mcr-2*, identified in *E. coli* isolates and sharing 76.7% of nucleotide identity with *mcr-1* (Liassine et al., 2016; Xavier et al., 2016); and *mcr-3*, identified in porcine *E. coli* isolates and sharing 45.0% of nucleotide sequence identity with *mcr-1* (Yin et al., 2017). These genes encode a phosphoethanolamine transferase family protein that modifies the lipid A component of LPS (Liassine et al., 2016; Yin et al., 2017) and confers a low level of colistin resistance (MIC = 4–8 mg/L) (Liu et al., 2016).

Of note, the *mcr-1* gene was found to co-occur with other antibiotic resistance genes, such as ESBL and NDM (Yang R. S. et al., 2016; Yang Y. Q. et al., 2016; Zheng et al., 2016), raising the likelihood of a serious bacterial infection that would be difficult to cure and contain. More importantly, a transposon element identified in *mcr-1*–harboring plasmids may expedite its mobilization to different plasmid backbones (Bernasconi et al., 2016; Doumith et al., 2016), and between bacterial strains and species, further propelling drug resistance in Gram-negative bacteria. Thus, far, the *mcr-1* gene has been found in different plasmid incompatibility (Inc) groups, including IncI2, X4, F, HI2, and P (Fernandes et al., 2016; Sun et al., 2016; Yang Q. E. et al., 2016). In pHNSHP45, the first reported *mcr-1*–harboring plasmid, IS*Apl1* is inserted directly upstream of the *mcr-1* gene (Petrillo et al., 2016). However, IS*Apl1* is absent in several contigs on IncI2, IncX4, and IncP plasmids, while different plasmid backbones contain a horizontally-transferred 2.6-kb sequence nearly identical to *mcr-1-pap2* (Li et al., 2016).

Before the discovery of *mcr*, clinical polymyxin resistance mechanisms were reported to be associated with chromosomal mutations or IS sequence insertions. A qualitative modification of LPS involves a large panel of genes and operons, including genes and operons that are directly involved in LPS modifications, i.e., the *pmrC* and *pmrE* genes; regulatory genes involved in two-component systems, i.e., the *pmrAB* and *phoPQ* genes; and regulators of these two-component systems, i.e., the *mgrB* gene (also called *yobG*), whose product negatively regulates the PhoPQ system (Poirel et al., 2017). It has been suggested that PmrAB or PhoPQ two-component systems that are associated with LPS modification may play an important regulatory role in colistin or polymyxin B resistance in several Gram-negative pathogens (Adams et al., 2009; Schurek et al., 2009). Moreover, amino acid polymorphisms of PmrA and/or PmrB have been observed in clinical isolates of *Acinetobacter baumannii* and *K. pneumoniae* (Poirel et al., 2017). In addition, MgrB alteration is a common mechanism of colistin resistance among KPC-*K. pneumoniae* in clinical settings. Quesada et al. showed that mutations in PmrAB were associated with colistin or polymyxin B resistance in *E. coli* isolated from poultry and swine; they found two unique amino acid substitutions in colistin-resistant *E. coli* strains. Nevertheless, *mcr*-negative colistin-resistant clinical isolates of *E. coli* are rarely reported. Chromosomal mutations of the *pmrAB, phoPQ*, and *mgrB* genes that result in lipid A modification might be harbored by clinical isolates of *E. coli*, and could be responsible for their resistance.

In the current study, we performed molecular epidemiological surveillance of colistin resistance in clinical isolates of *E. coli*, not only in *mcr*-positive isolates but also in *mcr*-negative isolates, and determined the mechanisms of resistance. We report a high incidence of medical records detailing colistin-resistant *E. coli* in the hospital. We identified 40 (3.15%) colistin-resistant isolates, including 21 *mcr*-positive genetically diverse isolates that all harbored *mcr-1*, but not *mcr-2* or *mcr-3*; the *mcr-1* gene was carried on plasmids of various sizes (ca. 30–270 kb), which were all readily transferrable to a recipient strain *in vitro*. Earlier studies presented evidence for the transmission of *mcr* genes from animals to human (Brauer et al., 2016; Liu et al., 2016). Here, we discovered that *bla*_CTX−M−55_ was the most common ESBL gene among the ESBL-producing *mcr*-positive isolates, which is another indication of a possible transmission of colistin resistance from animal to human. Among the ESBL-positive isolates unambiguously shown to harbor *mcr*-positive plasmid replicon groups, we demonstrated the prevalence and high potential for horizontal transfer of IncX4 plasmids of similar size and with the same genetic structure of *mcr*-positive elements. More significantly, 47.5% (19/40) of colistin-resistant *E. coli* isolates did not possess *mcr*, while their minimal inhibitory concentrations (MICs) for colistin were higher than those of an *E. coli* trans-conjugant harboring an *mcr*-positive plasmid. In our study, although five unique amino acid substitutions were identified, four in PmrB and one in PhoQ, two isolates did not have amino acids in PmrAB, PhoPQ, or MgrB that differed from those in sensitive isolates, indicating that other novel mechanisms were responsible for the colistin resistance of these isolates. Furthermore, the core genome phylogeny tree revealed that the *mcr*-positive and *mcr*-negative isolates were genotypically unrelated.

To the best of our knowledge, this study is the first-ever to report that a similar incidence of *mcr*-negative and *mcr*-positive colistin-resistant isolates (47.5 and 52.5%, respectively), with no demonstrated nosocomial transmission. In addition, we also report for the first time the molecular epidemiological surveillance for colistin resistance of *mcr*-negative *E. coli* clinical isolates as well as the mechanisms responsible for this resistance.

## Materials and methods

### Patients and bacterial isolates

In total, 1,270 *E. coli* isolates were obtained from the First Affiliated Hospital (Zhejiang University, Hangzhou, China) between January, 2014, and March, 2015. The patients were from different counties. The isolates were identified using the MALDI-TOF technique. The work was compliant with the ethical guidelines of the Declaration of Helsinki, and written informed consent was obtained from all patients prior to enrollment in this study. All procedures involving human subjects were approved by the medical ethics committee of the First Affiliated Hospital (College of Medicine, Zhejiang University).

### Clinical data and MIC testing

Patients who harbored colistin-resistant *E. coli* all dwell in Zhejiang Province; none of the patients were treated with colistin as an antimicrobial drug and none of the patients has ever traveled abroad (Table 1). Susceptibility testing to a wide range of antibiotics was routinely undertaken or performed using Vitek II (2017 CLSI). Colistin susceptibility was screened via the agar dilution method to determine MICs (Dafopoulou et al., 2015). The ESBL phenotype was confirmed by a standard double disc synergy test, as recommended by the CLSI. *E. coli* ATCC 25922 and *K. pneumoniae* ATCC 700603 were used as quality controls.

**Table 1:** Clinical features of colistin resistant *E. coli*.

**Isolate**	**Age**	**Sex**	**Source**	**Diagnosis**	**Job**	**Ward**	***mcr***
CoR-1	77	M	Blood	Biliary infection	Farmer	6B-16	+
CoR-2	69	F	Urine culture	Urinary infection	Farmer	10-5	+
CoR-3	56	F	Urine culture	Urinary infection	Retired	3-3	+
CoR-4	84	M	Urine culture	Urinary infection	Worker	5-7	–
CoR-5	31	M	Fester culture	Perianal infection	Pressman	2-11	+
CoR-6	63	M	Blood	Septicemia	Worker	ED	–
CoR-7	65	M	Fester culture	Pelvic abscess	Farmer	3-4	+
CoR-8	60	M	Fester culture	Pelvic abscess	Farmer	3-5	+
CoR-9	46	F	Urine culture	Urine infection	Worker	10-1	–
CoR-10	76	M	Blood	Septicemia	Farmer	EICU	+
CoR-11	66	M	Ascites	Abdominal infection	Retired	6A-4	+
CoR-12	81	M	Urine culture	Urinary infection	Farmer	3-4	–
CoR-13	58	F	Sputum culture	Pulmonary infection	Worker	ED	–
CoR-14	58	F	Sputum culture	Pulmonary infection	Worker	ED	–
CoR-15	53	M	Urine culture	Urinary infection	Farmer	10-1	–
CoR-16	71	F	Urine culture	Urine infection	Farmer	10-1	+
CoR-17	28	F	Urine culture	Urine infection	Worker	6B-9	+
CoR-18	59	F	Ascites	Gastric cancer	Farmer	5-1	–
CoR-19	93	M	Urine culture	Urine infection	Retired	6B-21	–
CoR-20	33	M	Sputum culture	Pulmonary infection	Worker	5-9	+
CoR-21	59	M	Blood	Obstructive cholangitis	Farmer	6B-16	+
CoR-22	93	M	Urine culture	Urine infection	Retired	6B-21	–
CoR-23	75	M	Urine culture	Urine infection	Worker	3-4	+
CoR-24	53	F	Sputum culture	Pulmonary infection	Farmer	ED	–
CoR-25	55	F	Ascites	Surgery infection	Farmer	6B-17	+
CoR-26	61	M	Sputum culture	Pulmonary infection	Farmer	2-19	–
CoR-27	61	M	Ascites	Gastric cancer	Worker	6B-11	+
CoR-28	60	F	Urine culture	Urine infection	Farmer	9-4	+
CoR-29	32	F	Urine culture	Urine infection	Farmer	10-1	–
CoR-30	56	M	Ascites	Gastric cancer	Farmer	ED	–
CoR-31	72	F	Urine culture	Urine infection	Farmer	6A-12	+
CoR-32	53	F	Ascites	Surgery infection	Worker	5-3	–
CoR-33	78	M	Urine culture	Urine infection	Farmer	3-3	+
CoR-34	66	M	Bile culture	Biliary tract infection	Retired	6B-12	–
CoR-35	76	M	Ascites	Abdominal infection	Retired	7-2	–
CoR-36	75	F	Ascites	Abdominal infection	Farmer	6B-12	–
CoR-37	43	F	Urine culture	Urine infection	Farmer	9-4	+
CoR-38	82	M	Urine culture	Urine infection	Retired	10-5	+
CoR-39	71	F	Bile culture	Biliary tract infection	Farmer	6B-16	–
CoR-40	42	F	Urine culture	Urine infection	Farmer	9-4	+

### PCR amplification, amino acid variants and MLST

The *mcr*-harboring isolates were screened by PCR (using primers for *mcr-1, mcr-2*, and *mcr-3*; Table S1), and were validated by sequencing. The entire *pmrAB, mgrB*, and *phoPQ* genes were amplified and sequenced using specific oligonucleotide primers listed in the appendix (Table S1). Amino acid sequences of *mcr*-negative colistin-resistant isolates were compared with those of colistin-sensitive clinical isolates, and the reference strain *E. coli* K-12 MG1655. Sorting Intolerant From Tolerant (SIFT) scores (http://sift.jcvi.org) were calculated to predict whether amino acid substitutions in MgrB, PmrAB, and PhoPQ affect protein function. In addition, SMART analysis (http://smart.embl.de/) was performed to determine the domain architectures of PmrA, PmrB, PhoP, PhoQ, and MgrB. *E. coli* MLST was performed using the Warwick database (http://mlst.warwick.ac.uk/mlst/dbs/Ecoli). PCR amplification was employed to detect β-lactamase genes. The primers for PCR detection and sequencing were as described in a previous study (Zhang et al., 2016). The basic local alignment search tool (BLAST) was used to analyze the sequencing results.

### Plasmid analysis, southern hybridization, and PFGE

Conjugation assays were performed largely according to a method described previously (Wang et al., 2003). *mcr-1*–harboring *E. coli* was used as the donor, while *E. coli* J53 (sodium azide-resistant) served as the recipient strain. Trans-conjugants were selected on Mueller-Hinton agar supplemented with sodium azide (100 mg/L) and colistin (2 mg/L). PCR and DNA sequencing were used to detect the presence of *mcr-1* gene in trans-conjugants; the colistin MICs of the conjugants were then determined. For the transformation experiments, plasmid DNA from *mcr*-negative isolates was prepared by alkaline lysis; the plasmid DNA was then electroporated into *E. coli* strain DH5α, and the bacteria plated on media containing 2 μg/mL of colistin.

To estimate the sizes of *mcr-1* plasmids, S1-PFGE and Southern hybridization were performed. Briefly, bacterial whole cell DNA was prepared in agarose plugs and digested with S1 nuclease (TaKaRa, Dalian, China). The DNA was separated using the CHEF-MAPPER PFGE system (Bio-Rad) under the following conditions: 14°C, 6 V/cm, and a 120° pulse angle for 16 h, with the initial and final pulses conducted for 2.16 and 63.8 s, respectively. The separated DNA fragments were transferred to nylon membranes, hybridized with digoxigenin-labeled *mcr-1*–specific probes (primer *mcr-1*-SB-F/R; Table S1) and detected using the NBT/BCIP color detection kit (Roche, cat. no. 11745832910).

PFGE of *mcr*-positive isolates and their trans-conjugants were performed to exclude the occurrence of spontaneous sodium azide resistant mutants. Briefly, whole cell DNA of clinical strains and markers (*Salmonella enterica* serovar Braenderup H9812) in agar gels were digested by XbaI and then separated by pulsed field electrophoresis, under the same conditions as S1-PFGE.

### Whole genome sequencing and bioinformatics analysis

Crude DNA extracts were prepared using QIAamp DNA mini kit (QIAGEN, cat. no. 51304) from overnight cultures of *E. coli*. The DNA was sequenced using the Illumina Hi-seq platform after library preparation. The quality-trimmed raw sequence data were assembled using Velvet 1.2.7. Prokka v 1.10 was used for the annotation of plasmid genes; PlasmidFinder (https://cge.cbs.dtu.dk/services/PlasmidFinder/) was used to detect plasmid Inc groups; Parsnp (https://github.com/marbl/parsnp) was used to align the core genome. The resistance genes were identified by performing BLASTn against the ResFinder 2.1 database (https://cge.cbs.dtu.dk/services/ResFinder/). Bioinformatics tools used in this work are available at the following web platforms: NCBI [National Center for Biotechnological Information, i.e., BRIG (BLAST Ring Image Generator)], SMS (Sequence Manipulation Suite), and EBI (European Bioinformatics Institute).

### Nucleotide sequence accession numbers

The whole genome sequences described in this paper have been deposited in DDBJ/ENA/GenBank under the accession numbers MKFK00000000-MKFS00000000, MKIH00000000-MKIL00000000, and IRU00000000- NISB00000000, and were in a processing queue (BioProject: PRJNA344524). The versions described in this paper are MKFK01000000-MKFS01000000, MKIH01000000-MKIL01000000, and NIRU00000000-NISB00000000.

**Table:** 

**Accession**	**Name**
MKFK00000000	CoR-1
MKFL00000000	CoR-2
MKFM00000000	CoR-3
NIRU00000000	CoR-4
MKIJ00000000	CoR-11
NIRV00000000	CoR-13
NIRW00000000	CoR-14
MKFR00000000	CoR-16
MKFN00000000	CoR-17
NIRX00000000	CoR-19
MKFO00000000	CoR-20
MKFP00000000	CoR-23
MKFQ00000000	CoR-27
MKFS00000000	CoR-28
NIRY00000000	CoR-29
NIRZ00000000	CoR-30
MKIH00000000	CoR-33
NISA00000000	CoR-35
NISB00000000	CoR-36
MKII00000000	CoR-37
MKIK00000000	CoR-38
MKIL00000000	CoR-40

## Results

### Detection of colistin-resistant clinical isolates and their clinical characteristics

From 1,270 clinical *E. coli* isolates, we identified 40 isolates that exceeded the colistin resistance breakpoint (>2 mg/mL) using the agar dilution method (Table 1). Of these, 21 *mcr*-positive isolates were identified using the *mcr-1–*specific primers and were clearly colistin resistant. Full gene sequencing confirmed that all these 21 strains encoded MCR-1. The other 19 colistin-resistant isolates were found to have no *mcr-1, mcr-2*, or *mcr-3* genes using specific primers (Table S1). These *mcr*-negative isolates had higher colistin MICs than those of the *mcr*-positive isolates (Table 2).

**Table 2:** Antibiotic susceptibilities (mg/L), sequence types (ST), ESBL genes, and *mcr-1*-harboring plasmid replicon types of *E. coli* isolates.

**Isolate**	**Antibiotics**											**Isolates/Conjugants Colistin MIC (mg/L)**	**ST**	**ESBL**	***mcr-1* plasmid replicon type**
	**AMP**	**AMK**	**CAZ**	**CIP**	**CST**	**CPC**	**GEN**	**IPM**	**LVX**	**SMX**	**TGC**				
CoR-1	>32	≤2	16	≥4	8	16	≤1	≤1	≥8	≥320	≤0.25	8/8	ST156^*^	CTX-M-55, OXA-10	IncI2
CoR-2	>32	≤2	8	≥4	4	2	≤1	≤1	≥8	≥320	≤0.25	4/4	ST10^*^	CTX-M-55	IncX4
CoR-3	>32	≤2	≤1	2	4	≤1	≥16	≤1	1	≥320	≤0.25	4/4	ST2253	CTX-M-14	IncF
CoR-4	≤ 2	≤2	≤1	0.2	>32	≤1	≤1	≤1	= 0.2	≤20	≤0.25	>32	ST101	-	-
CoR-5	>32	>64	>64	≥4	8	≥64	≥16	≤1	≥8	≥320	≤0.25	8/8	ST405	-	-
CoR-6	>32	≤2	16	0.2	32	≤1	≤1	≤1	1	≤20	≤0.25	32	ST101	-	-
CoR-7	>32	≤2	16	= 0.2	4	≥64	≤1	≤1	0.2	≤20	≤0.25	4/4	ST10^*^	CTX-M-55	IncI2/ IncF^a^
CoR-8	>32	≤2	16	= 0.2	4	≥64	≤1	≤1	0.2	≤20	≤0.25	4/4	ST10^*^	CTX-M-55	IncI2/ IncF^a^
CoR-9	>32	≤2	≤1	≥4	32	≤1	≤1	≤1	≥8	≤20	≤0.25	32	ST346	CTX-M-14	-
CoR-10	8	≤2	≤1	= 0.2	4	≤1	≤1	≤1	0.2	≤20	≤0.25	4/4	ST2598	-	-
CoR-11	>32	≤2	8	≥4	4	2	≥16	2	≥8	≥320	≤0.25	4/4	ST46	CTX-M-55	IncX4
CoR-12	>32	≤2	16	≥4	>32	8	≤1	≤1	≥8	≤20	≤0.25	>32	Novel	-	-
CoR-13	>32	≤2	>64	≥4	16	≥64	≤1	≤1	2	≥320	0.5	>32	ST14	CTX-M-55	-
CoR-14	>32	≤2	>64	≥4	32	≥64	≤1	≤1	≥8	= 320	0.5	>32	ST14	CTX-M-55	-
CoR-15	>32	>64	16	0.5	32	4	≥16	≤1	1	≥320	0.5	32	ST456	CTX-M-55	-
CoR-16	>32	8	≤1	≥4	8	2	≥16	≤1	≥8	>320	≤0.25	8/8	ST167	CTX-M-14	IncX4
CoR-17	>32	≤2	≥64	≥4	4	≥64	≥16	≤1	≥8	≥320	≤0.25	4/4	ST1011	CTX-M-55,CTX-M-64	IncF
CoR-18	≤ 2	≤2	≤1	0.2	32	≤1	≤1	≤1	0.2	≤20	0.5	32	ST2236	-	-
CoR-19	>32	4	4	≥4	>32	≥64	≥16	≤1	≥8	≥320	0.5.	>32	ST131	CTX-M-14	-
CoR-20	>32	8	≤1	≥4	4	2	≥16	≤1	≥8	≥320	≤0.25	4/4	ST410^*^	CTX-M-14, OXA-1	IncX4
CoR-21	≤ 2	≤2	≤1	≥4	4	≤1	≥16	≤1	≥8	≥320	≤0.25	4/4	ST88^*^	-	-
CoR-22	>32	4	8	≥4	>32	≥64	≥16	≤1	≥8	≥320	0.5	>32	ST70	-	-
CoR-23	>32	≤2	≤1	2	4	4	≥16	≤1	4	≥320	≤0.25	4/4	ST155	CTX-M-14, OXA-1	IncF
CoR-24	>32	≤2	≤1	0.2	>32	≤1	≥16	≤1	0.2	≥320	0.5	16	ST104	-	-
CoR-25	8	≤2	≤1	≥4	>16	≤1	≤1	≤1	≥8	≥320	≤0.25	>16/4	ST131^*^	-	-
CoR-26	>32	≤2	16	≥4	>32	≥64	≤1	≤1	≥8	≤20	0.5	32	ST165	CTX-M-55	-
CoR-27	>32	4	≤1	≥4	4	2	≥16	≤1	≥8	≥320	≤0.25	4/4	ST3944	CTX-M-3	IncX4
CoR-28	>32	≤2	16	≥4	8	16	≤1	≤1	≥8	≤20	≤0.25	8/8	ST457	CTX-M-55	IncI2
CoR-29	>32	≤2	2	0.2	>32	8	≤1	≤1	1	≤20	0.5	>32	ST38	CTX-M-14	-
CoR-30	>32	≤2	≥64	≥4	>32	16	≥16	≤1	≥8	≥320	0.5	>32	ST10	CTX-M-15	-
CoR-31	>32	≤2	≤1	≥4	4	≤1	≤1	≤1	2	≤20	≤0.25	4/4	Novel	-	-
CoR-32	>32	≤2	≥64	≥4	4	2	8	≤1	2	≤20	≤0.25	32	ST10	-	-
CoR-33	>32	≤2	≤1	≥4	8	≤1	8	≤1	≥8	≥320	≤0.25	8/8	ST206	CTX-M-14	IncF/IncX1^a^
CoR-34	>32	≤2	4	0.2	16	2	≤1	≤1	0.2	≤20	≤0.25	16	ST359	CTX-M-55	-
CoR-35	>32	≤2	4	≥4	16	2	≥16	≤1	≥8	≤20	≤0.25	>32	ST410	CTX-M-14	-
CoR-36	>32	4	4	≥4	>32	8	≥16	≤1	≥8	≤20	0.5	>32	ST354	CTX-M-14	-
CoR-37	>32	≤2	16	≥4	4	≥64	≥16	≤1	≥8	≥320	≤0.25	4/4	ST410^*^	CTX-M-55	IncFIC/IncFIB^a^
CoR-38	>32	≤2	16	≥4	4	4	≥16	≤1	≥8	≥320	≤0.25	4/4	ST206	CTX-M-55	IncFII/IncI2^a^
CoR-39	>32	4	≤1	≥4	32	≤1	≤1	≤1	≥8	≤20	0.5	32	ST749	CTX-M-14	-
CoR-40	>32	≤2	4	≥4	8	4	≥16	≤1	≥8	≥320	≤0.25	8/8	ST10^*^	CTX-M-65, OXA-1	IncX4

AMP, ampicillin; AMK, amikacin; CAZ, ceftazidime; CIP, ciprofloxacin; CST, colistin; CPC, cefepime; GEN, gentamicin; IPM, imipenem; LVX, levofloxacin; SMX, sulfamethoxazole; TGC, tigecycline; ND, not determined;

* *STs have been reported in mcr-positive isolates before*.

a *Proper mcr-1 plasmid replicon type identified by replicon type PCR of the trans-conjugants*.

According to the epidemiological data of the 40 corresponding patients (Table 1), the patients ranged in age from 28 to 93 years. These samples obtained from culture included urine (*n* = 18), ascites (*n* = 8), blood (*n* = 4), sputum (*n* = 5), and other secretions (*n* = 5; Table 1). Sample sources were not distinguishably different between *mcr*-positive and -negative isolates. The isolates were obtained from in 16 wards and the patients were from multiple cities in the Zhejiang Province, China. Medical records were reviewed, and patient histories confirmed that they had not been prescribed polymyxin or traveled abroad previously. With the exception of two patients who died of abdominal infection and respiratory failure, all the other patients recovered. The patients received empirical antibiotic treatment during their hospitalization, including levofloxacin, amikacin, piperacillin/tazobactam, cefixime, imipenem, and meropenem.

### Characteristics of the antibiotic susceptibility, ESBL phenotypes, and sequence types (STs)

The MICs of the *mcr*-positive colistin-resistant isolates were 4–8 mg/L, except for CoR-25 (>16 mg/L) (Table 2). However, for the *mcr*-negative colistin-resistant isolates, the MICs were ≥16 mg/L, and for more than half of them (10/19), they were >32 mg/L (Table 2). Further, 28 of the 40 isolates (70%) were ESBL-positive, while none of them was carbapenem resistant. Only one strain, CoR-11, showed intermediate sensitivity to imipenem, while the rest of the isolates were susceptible. Gentamicin resistance was seen in 19/40 (47.5%) of the isolates, with two isolates displaying a high level of amikacin resistance (Table 2). High resistance ratios to ciprofloxacin (70%) and levofloxacin (62.5%) were seen in these isolates. The colistin resistant isolates retained susceptibility to tigecycline (Table 2). The observed antibiotic susceptibilities of *mcr*-positive and *mcr*-negative colistin-resistant isolates were not markedly different. Further, sequence analysis revealed that the ESBL genes were present in a wide variety of *E. coli* STs, and most ESBL-producing *E. coli* produced more than one type of β-lactamase. The most common ESBL gene was *bla*_CTX−M−55_ in the CTX-M-1 group (14/28, 50.0%), followed by *bla*_CTX−M−14_ (11/28, 39.3%) in the CTX-M-9 group.

MLST differentiated the 21 representative *mcr*-positive *E. coli* strains into 15 STs and one unknown ST (untypable). The most common ST was ST10 (*n* = 4), followed by ST410 (*n* = 2), and ST206 (*n* = 2), and then by single ST type isolates. MLST also differentiated the *mcr*-negative colistin-resistant isolates into many different STs, with no obvious dominant ST (Table 2). PFGE analysis of the colistin-resistant isolates was also performed to evaluate any clonal relationships between the *mcr*-positive and *mcr*-negative resistant strains. PFGE indicated that the isolates were not genetically related, and not nosocomially transmitted (Figure S1). Hence, colistin-resistant clinical isolates comprised a variety of STs and were therefore genetically different, with the nosocomial transmission excluded.

### Plasmid analysis and southern blotting

All plasmids harboring the *mcr-1* gene were transferable to *E. coli* J53. The trans-conjugants were resistant to colistin, with the MIC of 4 or 8 mg/L, which was consistent with their mother strains, except for CoR-25 (4 mg/L, Table 2). The MIC of the CoR-25 conjugant was lower than that of CoR-25, a finding that might indicate that chromosomal PhoQ mutation contributed to the colistin resistance. In order to exclude the occurrence of spontaneous sodium azide resistant mutants in the trans-conjugants, PFGE analysis of *mcr*-positive isolates vs. the recipient cells were performed (Figure S1). The results indicated that the trans-conjugants were all from the recipient cells.

**Figure 1:**
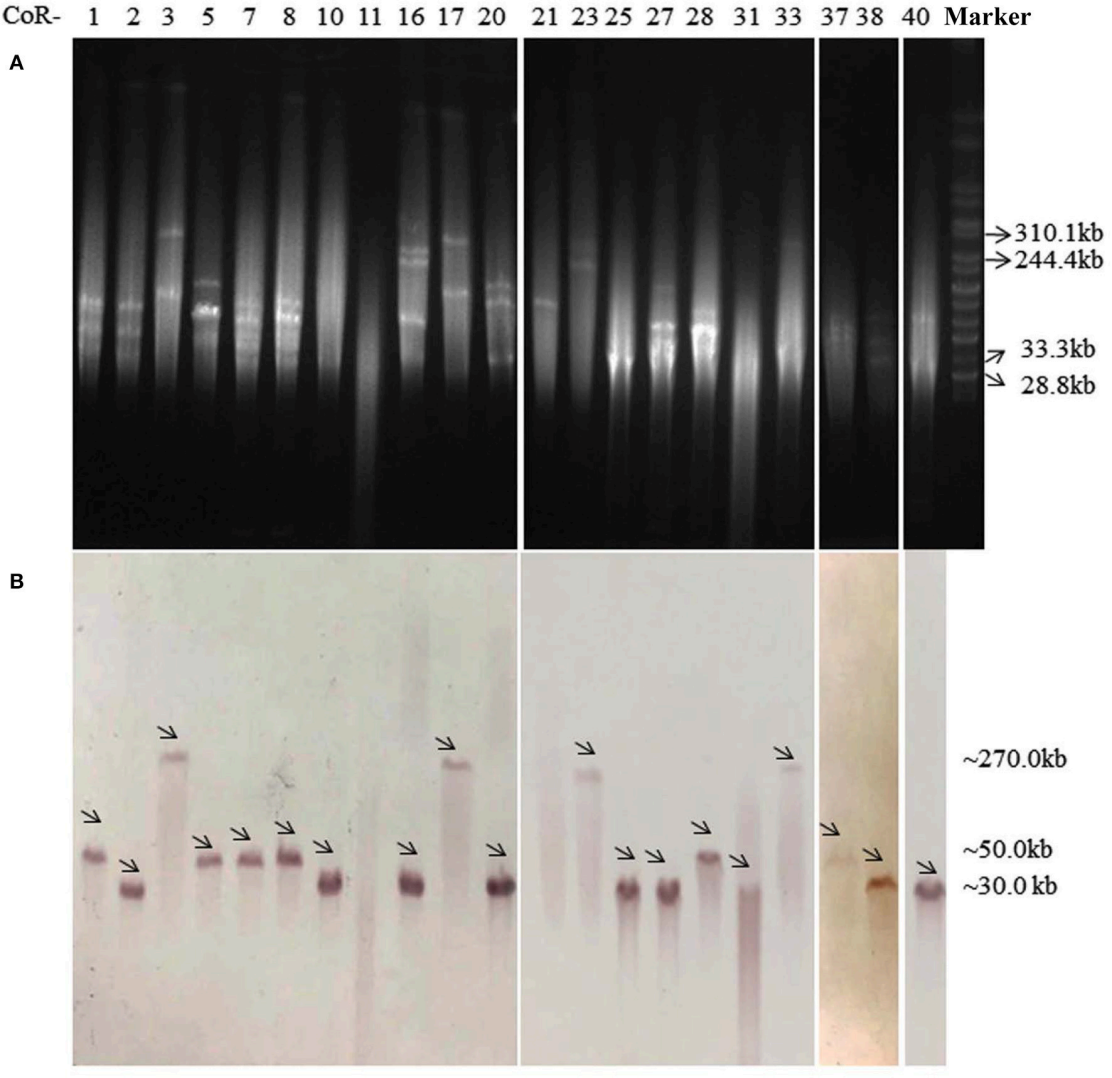
S1-PFGE profiles **(A)** and Southern blot **(B)** analysis with an *mcr-1–*specific probe of 21 *mcr-1*–harboring *E. coli* isolates, with *Salmonella enterica* serovar Branderup as a molecular mass marker (the last lane). The names of the isolates (“CoR-”) were shown in the first line of **(A)**. The arrows in **(B)** indicated the locations of *mcr-1*-harboring plasmids according to Southern blot experiment. The *mcr-1* Southern blot hybridization primers (*mcr-1*-SB-F/R) are shown in Table S1.

On the basis of the total DNA S1-PFGE and corresponding Southern hybridization, we determined that the *mcr-1* gene was exclusively located on diverse plasmids, with plasmid sizes ranging from ca. 30 to 270 kb (Figure 1). Southern hybridization of CoR-11 and CoR-21 was unsuccessful for unknown reasons; however, the presence of *mcr-1* was confirmed by PCR and the strains' *mcr*-positive plasmids were transferable and conferred colistin resistance. Further, 16 of the 21 *E. coli* isolates that carried *mcr-1* were ESBL-positive (Table 2), and 14 of them were selected for whole genome sequencing. Whole genome analysis revealed three replicon types (IncX4, IncF, and IncI2) in the ESBL-producing isolates, which unambiguously confirmed *mcr-1*–harboring plasmid replicon groups (Table 2). IncX4 accounted for more than half of these plasmids (6/11), indicating the prevalence of *mcr-1*–harboring IncX4 in Zhejiang, China.

### The genetic structure of *mcr-1* in the *mcr*-positive isolates

**Figure 2:**
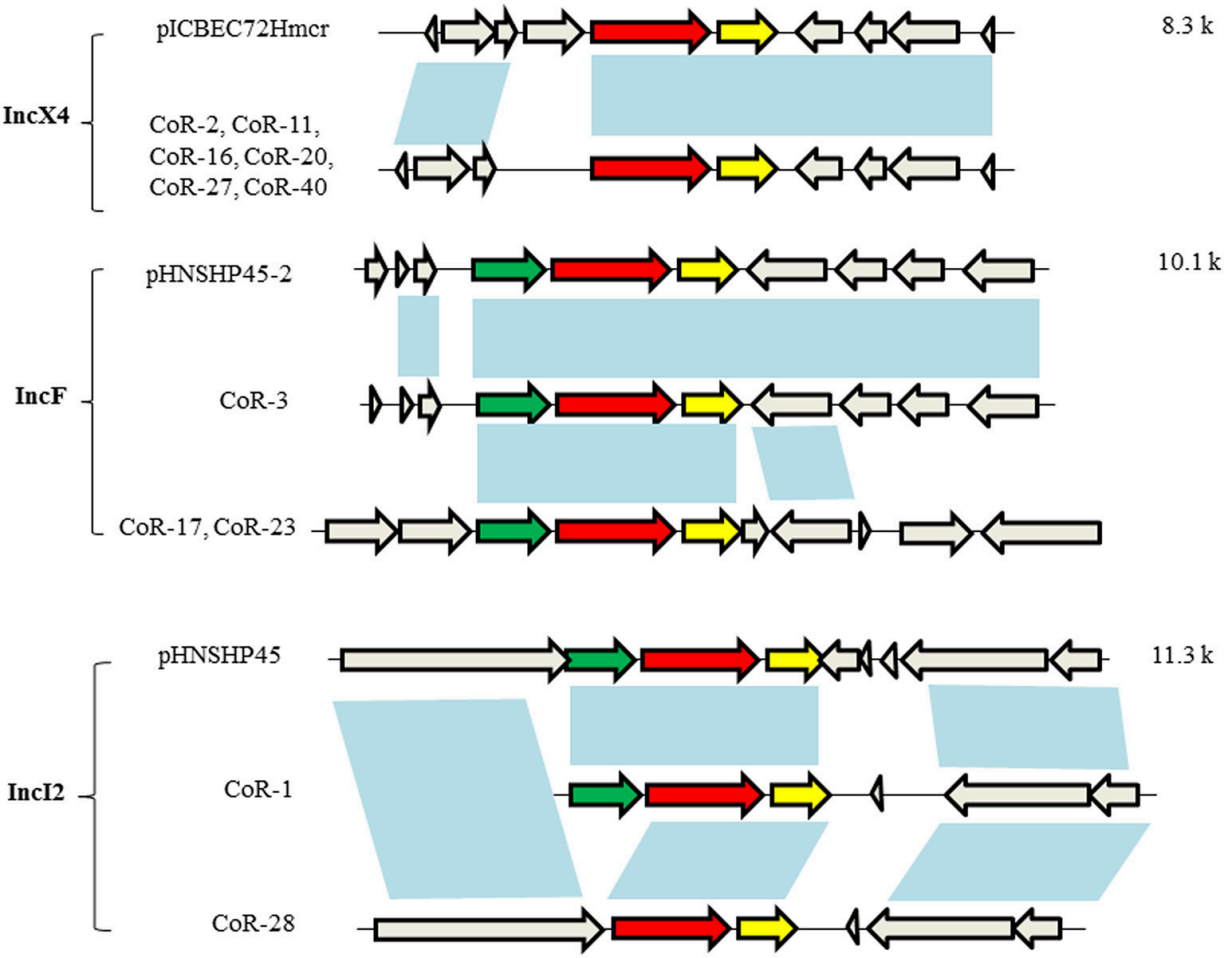
Genetic structures of the *mcr-1*–harboring elements of selected plasmids. Plasmid incompatibility groups are noted on the left, along with the plasmids and/or strains harboring the depicted genetic structure. Sequence lengths of the depicted areas are noted on the right. The arrows denote open reading frames (ORFs), with the red, yellow, green, and gray arrows denoting *mcr-1, pap2*, IS*Apl1*, and the neighboring genes, respectively. The blue shading indicates the same ORF.

The open reading frames (ORFs) in *mcr-1*–harboring plasmid contigs (ca. 9.3 kb) from CoR-2, CoR-11, CoR-16, CoR-20, CoR-27, and CoR-40 were all identical and belonging to the IncX4 incompatibility group. Sequence alignment revealed that these contigs were similar to those of other IncX4 plasmids, including pICBEC72Hmcr (Fernandes et al., 2016), from clinical isolates in Brazil, and pESTMCR (Brauer et al., 2016), isolated from a pig slurry in Estonia (Figure 2). The Southern hybridization data indicated that these IncX4 plasmids were all ca. 30 kb in length, suggesting that they were possibly from the same plasmid. The IncX4 plasmid was present in six phylogenetically different STs *E. coli* isolates, suggesting horizontal transfer, and no obvious ST preference. Interestingly, the insertion element IS*Apl1*, initially found to be associated with *mcr-1* in pHNSHP45, was not present in IncX4 plasmids in the current study.

The *mcr-1*–harboring plasmid contigs from CoR-3, CoR-17, and CoR-23 belonged to the IncF group, i.e., the same plasmid incompatibility group as pHNSHP45-2. Sequence alignment indicated that the ca. 160-kb contig of the CoR-3 IncF plasmid shared 99% identity with pHNSHP45-2 (Figure 2). A comparison of plasmids from CoR-3, CoR-17, and CoR-23, and the reported plasmid pHNSHP45-2 is shown in Figure S2. Moreover, the ca. 177-kb contig from the CoR-17 IncF plasmid and the ca. 97-kb contig from the CoR-23 IncF plasmid were identical. In addition, IS*Apl1* was present in IncF plasmids (Figure 2). The *mcr-1*–harboring plasmid contigs from CoR-1 and CoR-28, belonging to the IncI2 group, were the same in the plasmid incompatibility group as pHNSHP45. A comparison of the *mcr-1* genetic structure of CoR-1 and CoR-28 with that of pHNSHP45 revealed ORF differences (Figure 2, Figure S3). The *mcr-1*–associated IS*Apl1* element was not detected in the IncI2 plasmid from CoR-28 but it was present in the IncI2 plasmid from CoR-1 (Figure 2). Most importantly, an approximately 2.6-kb *mcr-1*–*pap2* element was found to be shared by all *mcr-1*–harboring plasmids.

### Amino acid variations of PmrAB, PhoPQ, and MgrB in *mcr*-negative isolates

To determine the exact colistin resistant mechanism in *mcr*-negative resistant isolates, we first had to eliminate the transferable resistant genes mediating colistin resistance. Therefore, conjugation and transformation experiments were done using the *mcr*-negative resistant isolates. We found that colistin-resistant phenotypes of all these strains were not transferable to *E. coli* J53. In the absence of a plasmid bearing the *mcr* gene, the *mcr*-negative colistin-resistant isolates may have acquired colistin resistance via chromosomal mutations in the *mgrB, phoPQ*, and, *pmrAB* genes that might confer lipid A modifications.

We next determined whether PmrAB, PhoPQ, and MgrB mutations conferred colistin resistance in the 10 *mcr*-negative isolates with high-MIC values (MICs > 32 mg/L). We randomly selected nine colistin-sensitive *E. coli* clinical isolates (S1–S9, Table 3) as negative controls, and used K-12 MG1655 as a reference. Many nucleotide variations were found, but most of them were synonymous; amino acid alterations were found at four sites in PmrA; 9 sites in PmrB; one site in PhoP; six sites of PhoQ; and one site of MgrB. While 11 amino acid substitutions were found in both colistin-resistant and sensitive isolates, only five amino acid substitutions were identified in the 10 *mcr*-negative resistant isolates with high MICs (Table 3). Most of the amino acid substitutions unique to the high-MIC isolates were found in PmrB; these were A118T, E123D, Y315F, and Y358N, located in the histidine kinases phosphatases domain (HAMP) and histidine kinase-like ATPases domain (HATPase_c), according to SMART analysis. N346K in the HATPase_c domain of PhoQ was also identified in one colistin-resistant isolate.

**Table 3:** Amino acid variations in functional domains of PmrAB, PhoPQ, and MgrB in clinical *E. coli* isolates.

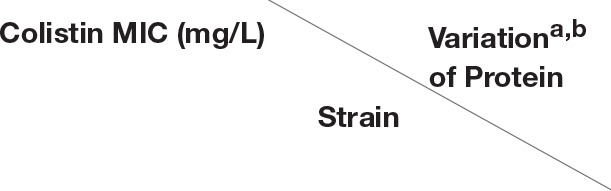			**PmrA**				**PmrB**									**PhoP**	**PhoQ**						**MgrB**	
			**REC**		**Unkown**		**Trans_ reg**		**HAMP**		**HATPase_c**					**REC**	**Trans_ reg**		**HATPase_c**				**Trans_ reg**	**Unkown**
			**29**	**31**	**128**	**144**	**2**	**19**	**118**	**123**	**283**	**315**	**351**	**358**	**360**	**44**	**6**	**138**	**386**	**346**	**467**	**482**	**8**	**41**
		**K-12 MG1655**	**S**	**T**	**I**	**G**	**H**	**G**	**A**	**E**	**D**	**Y**	**V**	**Y**	**A**	**I**	**R**	**S**	**V**	**N**	**L**	**A**	**V**	**I**
**R**	>32	CoR-4	G								G			**N**		L								
	>32	CoR-12	G																					
	>32	CoR-13	G	S	N	S	R	R		**D**	G		I			L							A	
	>32	CoR-14	G	S	N	S	R	R		**D**	G		I			L							A	
	>32	CoR-19	G	S			R			**D**	G		I			L							A	
	>32	CoR-22	G	S							G		I			L							A	
	>32	CoR-29	G				R				G	**F**				L								
	>32	CoR-30	G						**T**															
	>32	CoR-35	G			S					G			**N**										
	>32	CoR-36	G				R				G				V	L				**K**				
**S**	0.5	S-1	G				R				G				V	L	H						A	
	0.25	S-2	G				R												L					
	0.25	S-3	G																			T		
	0.25	S-4	G				R				G					L		T				T		
	0.5	S-5	G	S	N	S	R				G		I			L	H						A	
	0.5	S-6	G	S	N	S	R				G		I			L	H						A	L
	0.5	S-7	G				R				G					L		T				T		
	0.25	S-8	G				R				G				V	L					M			
	0.5	S-9	G																					

*^a^The predicted domains are indicated as follows: REC, cheY-homologous receiver domain; Unknown, Unknown domain; Trans_ reg, Transmembrane region; HAMP, histidine kinases phosphatases domain; HATPase_c, Histidine kinase-like ATPase domain; Only domains displaying substitutions are shown*.

*^b^Amino acid substitutions unique to mcr-negative colistin resistant isolates are indicated by bold type and underlined. Amino acids in green background are those to be predicted affect the function of the proteins by SIFT score (<0.05)*.

### Mutations in PmrB and PhoQ may contribute to the colistin-resistant phenotype of the *mcr*-negative isolates

Mutations in both PmrB and PhoQ might have affected phosphate transfer between the two system components since all of them were located in the kinase and phosphate-related domains. The E123D and Y315F substitutions in PmrA, and N346K in PhoQ were predicted to affect the function of proteins encoded by these genes, according to the SIFT score. However, no unique amino acid substitutions were identified in PmrA, PhoP, and MgrB in the 10 high-MIC value (MICs > 32 mg/L) colistin-resistant isolates, and neither frameshift mutations nor deletions were identified. Two of the 10 high-MIC value *mcr*-negative resistant isolates (CoR-12 and CoR-22) did not harbor amino acid variations in these proteins that could be used to differentiate them from the sensitive isolates. This indicated that a change in gene expression or other gene mutations contribute to colistin resistance in *E. coli*.

**Figure 3:**
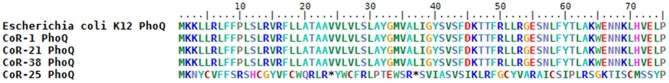
Partial sequence alignment of the PhoQ protein in CoR-25 and the control strains. The controls share the same amino acid sequence; CoR-25 PhoQ harbors a K to N substitution at position 3, which results in a premature stop codon at codon 24 and truncation of the PhoQ protein.

Interestingly, the *phoQ* allele of CoR-25 contained a frameshift mutation (deletion of A at nucleotide position 9) corresponding to a change of Lys to Asn, resulting in a premature stop at codon 24, with truncation of the PhoQ protein (Figure 3). This PhoQ frameshift mutation may have contributed to the high-level colistin resistance of the *mcr-1*–harboring isolate CoR-25.

### The *mcr*-positive and *mcr*-negative isolates are genotypically unrelated

**Figure 4:**
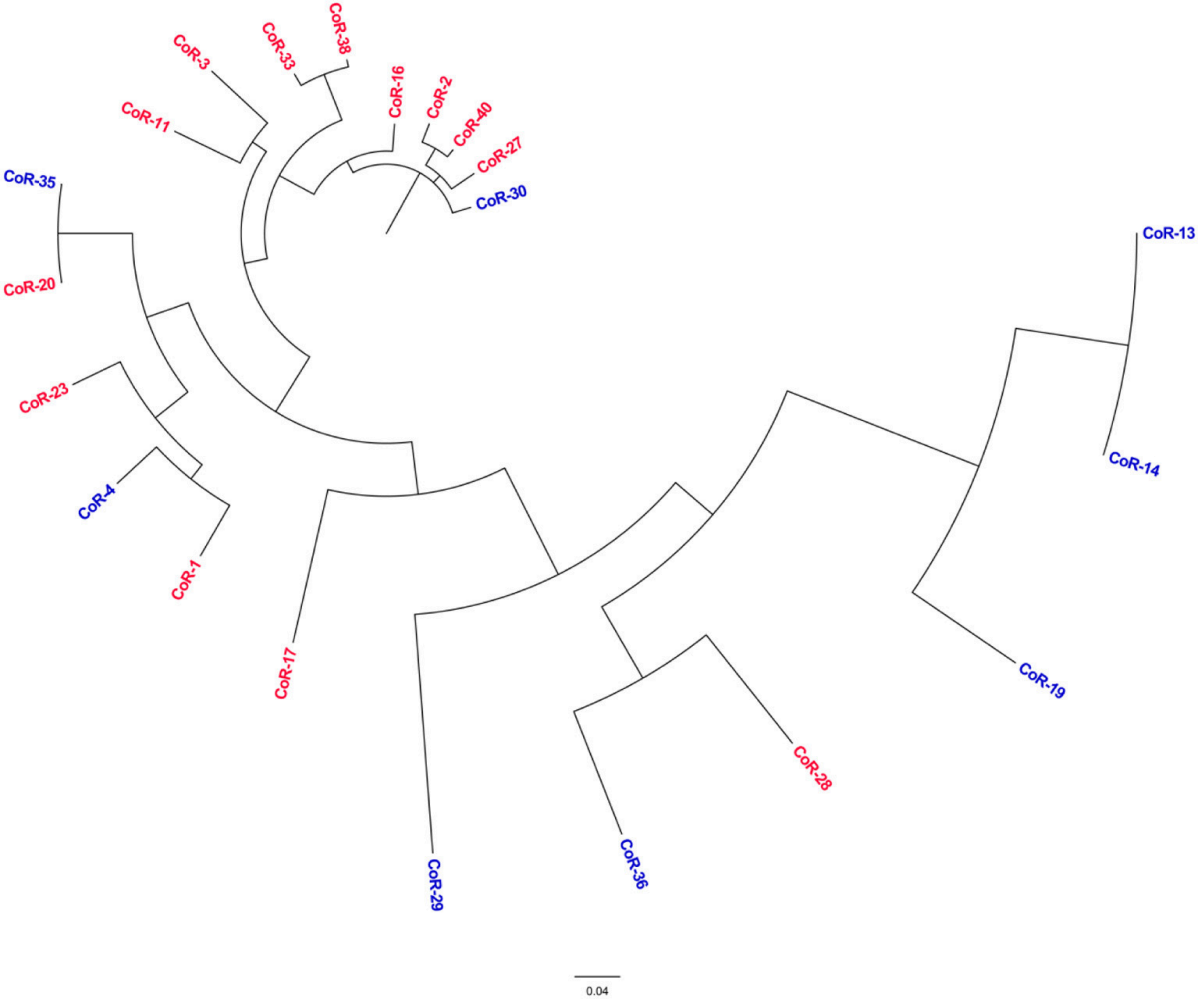
Phylogenetic tree analysis. The phylogeny of colistin-resistant clinical *E. coli* isolates based on the genotype at core genome SNPs. The characters in red correspond to the *mcr*-positive isolates, and in blue to the *mcr*-negative isolates.

Parsnp was employed to compare the core genomes of the two groups or isolates (the *mcr*-positive and *mcr*-negative colistin-resistant isolates). The final Parsnp multiple alignment contained all SNPs, Indel scores, and structural variations within the core genome; 13 *mcr*-positive colistin-resistant isolates and 8 *mcr*-negative colistin-resistant isolates were selected for core genome alignments and phylogeny reconstruction (one of the 14 sequenced *mcr*-positive isolate genomes was so remote from the genomes of these isolates that we could not add it to the phylogenetic tree). The core genome phylogeny tree revealed that the *mcr*-positive and *mcr*-negative isolates were genotypically unrelated (Figure 4). The *mcr*-negative isolates were remotely related while 8 of the 13 *mcr*-positive isolates were clustered together, indicating that *E. coli* backgrounds enabling the acquisition of *mcr*-harboring plasmids might be to some extent core genome-specific, which is an observation worthy of further study.

## Discussion

In China, where colistin is not used in the clinic for the treatment of infectious disease, the extensive use of antibiotics in food animal production, including swine and poultry farming, has unfortunately increased the risk of transfer of the resistant bacteria to human. In the current study, no patients had a history of colistin treatment. The spread of *mcr-1* might be associated with the occupations of the patients, as more than half of the *mcr-1*–harboring patients were farmers. In China, some farmers use colistin to increase the quality of food animals, which they raise in their backyards. More importantly, the five reported *mcr-1*–harboring STs identified in the current study (156, 10, 410, 88, and 131) had been previously isolated from poultry and swine (Bai et al., 2016; Ewers et al., 2016; Falgenhauer et al., 2016; Yang R. S. et al., 2016). This included ST10, the most prevalent ST in the current study, which has also been isolated from travelers returning from India (Bernasconi et al., 2016) and from European animals (El Garch et al., 2016). The ST diversity of the *mcr-1*–harboring *E. coli* isolates in the present study indicated a scattered and non-clonal prevalence of *mcr-1*–harboring bacteria. Furthermore, *bla*_CTX−M−55_ is the dominant CTX-M-1 group gene in *E. coli* isolated from the environment and swine in China (Hu et al., 2013), while the CTX-M-15 and CTX-M-14 genes are harbored by the majority of clinical *E. coli* isolates in China (Liu et al., 2015). The high incidence of CTX-M-55 in the *mcr-1*–harboring clinical isolates observed in the current study provides further evidence for the possible transmission between the human and animals, via a *mcr-1* gene–harboring pathogen or the gene itself. Therefore, the use of colistin could result in selective pressure in the farm environment and transmission of *mcr* genes to human.

Here, the genetic structures of the IncX4 contigs from six phylogenetically different strains were exactly the same, and the IncX4 plasmids had the same size, as determined by Southern hybridization. These findings may indicate that these plasmids have the same origin. However, the six isolates belonged to five different STs, implying high potential for horizontal transfer of the IncX4 plasmid. The discovery of *mcr-1* gene associated with IS*Apl1* on a conjugative plasmid may signify its transfer to different plasmid backbones, as well as between different bacterial species and genera. Of note, in the current study, a nearly 2.6-kb *mcr-1*–*pap2* element was found to be shared by all the sequenced *mcr-1*–carrying plasmids. Nevertheless, further investigation is needed to identify the *mcr-1* transfer mechanism. Interestingly, 8 of the 13 *mcr*-positive colistin-resistant isolates clustered together, indicating that *E. coli* backgrounds that favor the acquisition of *mcr*-harboring plasmids may have some core genome in common. This should be further investigated with a large sample size.

In this study, none of the patients had a history of colistin treatment. This might indicate that the increasing use of colistin or polymyxin B in agriculture, livestock, and aquaculture has increased polymyxin resistance acquired via diverse mechanisms, i.e., not only by a horizontal transfer of *mcr*-positive plasmids but also by chromosomal mutations that confer high-MIC resistance in the absence of *mcr*. Overall, the colistin resistance of the clinical isolates was not only associated with *mcr-1* but also with chromosomal mutations, although the patient histories confirmed that they had not previously received polymyxin treatment. It is of note that the *mcr*-negative colistin-resistant isolates were characterized by higher MICs than the *mcr*-positive isolates, posing more challenging in clinical treatment. The *mcr*-negative colistin-resistant isolates had higher MICs than the *mcr*-positive isolates, which will undoubtedly render clinical treatment more challenging. Although, chromosomal mutations cannot be horizontally transferred, the high incidence of *mcr*-negative colistin-resistant isolates cannot be ignored.

Mutations leading to the addition of cationic groups to lipid A result in a less anionic lipid A and changes leading to inactivation of lipid A biosynthesis result in a complete loss of LPS; consequently, this leads to reduced colistin binding or loss of the colistin target (Poirel et al., 2017). Several chromosomally encoded genes and operons are associated with colistin resistance in Gram-negative bacteria, such as PmrA/PmrB and PhoP/PhoQ two-component systems and the *mgrB* gene, which encodes a negative regulator of PhoPQ. The mechanisms of colistin resistance can be determined by sequencing these genes to detect mutations. Specific mutations in the PmrAB and PhoPQ two-component systems are associated with colistin resistance in some bacteria, e.g., *P. aeruginosa*. Inactivation of the MgrB regulator was reported to be associated with colistin resistance of *K. pneumoniae*. In the current study, we identified 19 highly colistin-resistant *mcr-1* negative isolates (MIC ≥16 mg/L). The high level of resistance detected among clinical isolates in this study suggests that the resistance was a consequence of chromosomal mutations. Quesada et al. showed that mutations in PmrAB are associated with colistin or polymyxin B resistance in *E. coli* isolated from poultry and swine. The authors found two unique amino acid mutations in colistin-resistant *E. coli* strains, R81S in PmrA and V161G in PmrB. In this study, we identified five amino acid substitutions unique to *mcr*-negative colistin-resistant isolates, four in PmrB and one in PhoQ. These mutations have not been identified in previous studies. Mutations in both PmrB and PhoQ might have affected phosphate transfer between the two system components since all of them were located in the kinase and phosphate-related domains.

Remarkably, since a loss-of-function mutation of *phoQ* can contribute to high-level polymyxin resistance in clinical strains (Miller et al., 2011), the PhoQ frameshift mutation might have contributed to the high colistin resistance of CoR-25, one of the *mcr-1*–harboring isolates that exhibited high colistin resistance. To the best of our knowledge, this is the first-ever description of a *mcr*-positive *E. coli* where a chromosome mutation most likely confers a high level of colistin resistance.

Although PmrAB and PhoPQ may be responsible for most polymyxin resistance in Gram-negative pathogens, the mechanisms of polymyxin resistance appear to be highly diverse. Among the 10 high-MIC *mcr*-negative resistant isolates, two isolates (CoR-12 and CoR-22) did not harbor amino acid variations in PmrAB, PhoPQ, or MgrB that could be used to differentiate them from the sensitive isolates. Thus, amino acid changes in PmrAB, PhoPQ, and MgrB might not be essential for colistin resistance in *E. coli*. However, many chromosomally-encoded mechanisms of resistance remain to be identified in *E. coli*. In *A. baumannii*, colistin resistance can be induced *in vitro* by serial passages in media containing increasing colistin concentrations and may be mediated by diverse mechanisms. However, it is difficult to be sure that the amino acid substitutions found in genes involved in LPS biosynthesis in clinical isolates with complicated genomic backgrounds lead to resistance. On the other hand, resistance could depend on the expression levels of the genes involved in colistin resistance in *E. coli*. Therefore, we explored the relative expression levels of *pmrAB, phoPQ*, and *mgrB* genes in 10 high-MIC *mcr*-negative resistant isolates and compared them with those of 9 *mcr*-negative colistin sensitive clinical isolates (S1–S9). Unfortunately, the expression levels varied considerably, and there were no differences that could be used to distinguish the colistin resistant isolates from the colistin sensitive isolates (data not shown). Thus, the gene expression changes that might contribute to colistin resistance in *E. coli* are difficult to determine in clinical isolates but not in the parental strain that underwent serial passages in media with increasing concentrations of colistin. Other mechanisms of colistin resistance are conceivable and will be investigated in a future study.

## Author contributions

Conceived and designed the experiments: QL, YX, and LL. Performed the experiments: QL, WY, CH, HX, PS, HL, and SX. Analyzed the data: QL, KZ, and LG. Wrote the paper: QL.

### Conflict of interest statement

The authors declare that the research was conducted in the absence of any commercial or financial relationships that could be construed as a potential conflict of interest.
