# Supplementary material for: Molecular Epidemiology and Colistin Resistant Mechanism of mcr-Positive and mcr-Negative Clinical Isolated Escherichia coli
Source: Front Microbiol. 2017 Nov 17;8:2262. doi: 10.3389/fmicb.2017.02262 (PMC5715374; doi:10.3389/fmicb.2017.02262)
Supplement: Supplementary file 1 [file DataSheet1.docx]

Supplementary Material

**Molecular Epidemiology and Colistin Resistant Mechanism of *mcr*-Positive and *mcr*-Negative Clinical Isolated *Escherichia coli***

Qixia Luo^1^, Wei Yu^1,2^, Kai Zhou^1^_,_ Lihua Guo^1^, Ping Shen^1^, Haifeng Lu^1^, Chen Huang^1^, Hao Xu^1^, Shaoyan Xu^3^, Yonghong Xiao^1*^, Lanjuan Li^1^.

^1^State Key Laboratory for Diagnosis and Treatment of Infectious Diseases; Collaborative Innovation Center for Diagnosis and Treatment of Infectious Diseases, the First Affiliated Hospital of Medical School, College of medicine, Zhejiang University, Hangzhou, China.

^2^Department of Infectious Diseases, Zhejiang Provincial People's Hospital, People's Hospital of Hangzhou Medical College, Hangzhou, China.

^3^Division of Hepatobiliary and Pancreatic Surgery, Department of Surgery, First Affiliated Hospital, School of Medicine, Zhejiang University, Hangzhou, China.

***Corresponding author**: Yonghong Xiao
E-mail addresses: [xiao-yonghong@163.com](mailto:xiao-yonghong@163.com)


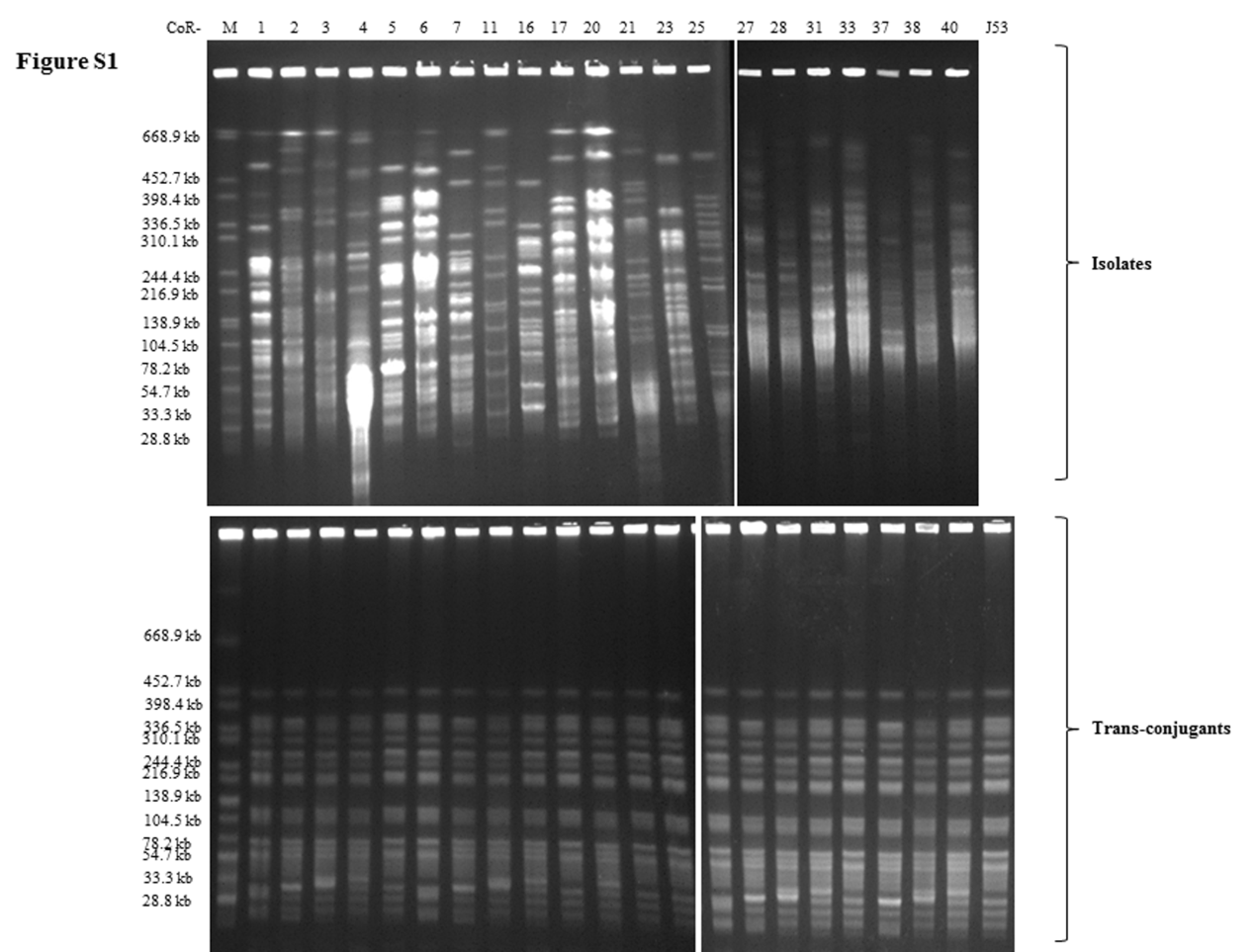


**Figure S1.** PFGE (Pulsed Field Gel Electrophoresis) analysis of the *mcr*-positive isolates and their trans-conjugants. Above: *mcr*-positive isolates, below: the corresponding trans-conjugants. Marker: Salmonella enterica serovar Braenderup H9812 digested by XbaI. Conditions: 14 °C, 6 V/cm, and 120° pulse angle for 16 h, with the initial and final pulses conducted for 2.16 and 63.8 s, respectively.

**
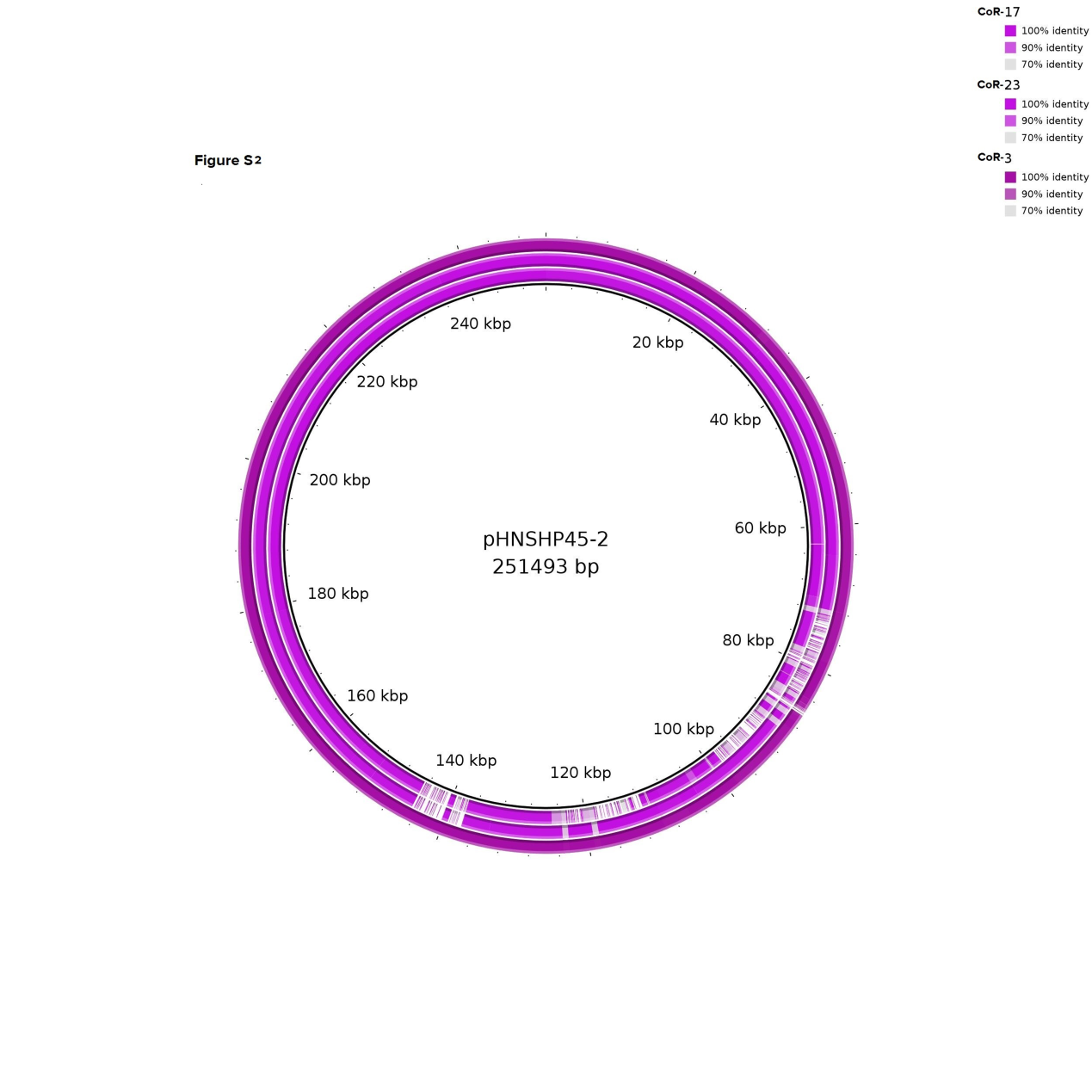
**

**Figure S2**. BRIG (BLAST Ring Image Generator) analysis of the *mcr*-positive plasmids from CoR-3, CoR-17 and CoR-23. Comparative analysis of *mcr*-positive plasmids from CoR-3, CoR-17 and CoR-23 with the reported plasmid pHNSHP45-2, a closely related *mcr*-1-harboring plasmids from *E. coli*. The concentric rings display similarity between the reference sequence in the inner ring and the other sequences in the outer rings. The color levels as shown to the right of the ring, indicate a matched degree of shared regions by BLAST result.


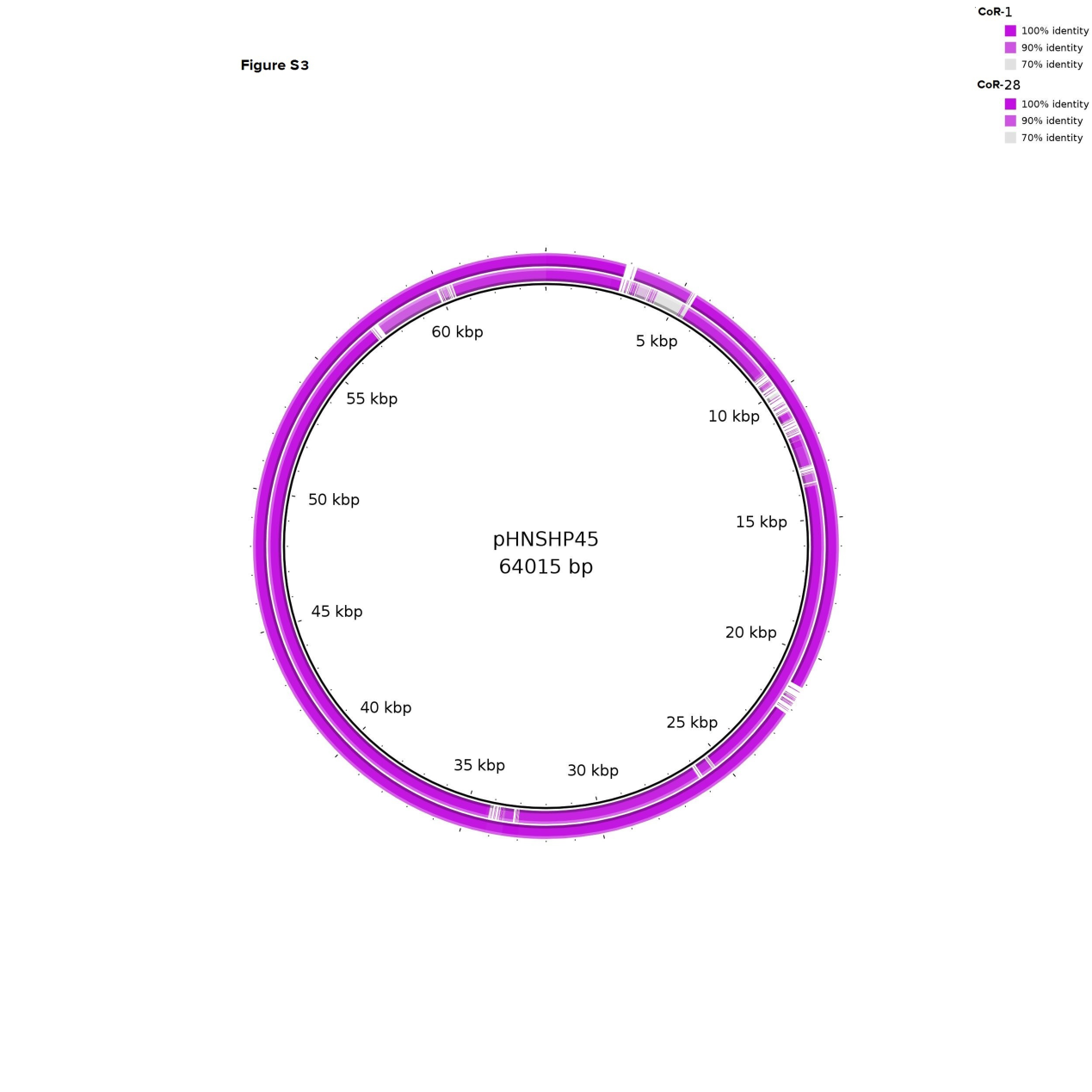


**Figure S3**. BRIG (BLAST Ring Image Generator) analysis of the *mcr*-positive plasmids from CoR-1 and CoR-28. Comparative analysis of *mcr*-positive plasmids from CoR-1 and CoR-28 with the reported plasmid pHNSHP45, a closely related *mcr*-1-harboring plasmids from *E. coli*. The concentric rings display similarity between the reference sequence in the inner ring and the other sequences in the outer rings. The color levels as shown to the right of the ring, indicate a matched degree of shared regions by BLAST result.

**Table S1** PCR primers used in this study.

| Primer name | Sequence | Product | Source |
| --- | --- | --- | --- |
| EC-*pmrA*-F | AGTTTTCCTCATTCGCGACCA | 714bp | Quesada et al., [^7^](#_ENREF_7) |
| EC-*pmrA*-R | TACCAGGCTGCGGATGATATTCT |  |  |
| EC-*pmrB*-F | GGATGGCCTGATGTGACGCTGTC | 1312bp | Quesada et al., [^7^](#_ENREF_7) |
| EC-*pmrB*-R | GCGCGGCTTTGGCTATA |  |  |
| EC-*mgrB*-F | CACGAATATCGACATAGTTAG | 275bp | This study |
| EC-*mgrB*-R | TATTCTACCACTGCTGGAGAG |  |  |
| EC-*phoP*-F | GCCAGTACCGCCAGCTTAA | 1798bp | This study |
| EC-*phoP*-R | CTCGCCACGTAACAGCCGAA |  | This study |
| EC-*phoQ*-F | GGCACAATATCCCCAAGAAGT | 1595bp | This study |
| EC*-phoQ*-R | ATCCACAGGCTGGTATCTGCA |  | This study |
| *mcr-1*-F | ATGATGCAGCATACTTCTGTG | 1626bp | This study |
| *mcr-1*-R | TCAGCGGATGAATGCGGTG |  | This study |
| *mcr-2*-F | ATGACATCACATCACTCTTGG | 1626bp | Liassine *et al*., 2016 |
| *mcr-2*-R | TTACTGGATAAATGCCGCGC |  |  |
| *mcr-3*-F | TTGGCACTGTATTTTGCATTT | 1626bp | Yin *et al*., 2016 |
| *mcr-3*-R | TTAACGAAATTGGCTGGAACA |  |  |
| *mcr-1*- SB-F | ATGCAGCATACTTCTGTGTGG | 776bp | This study |
| *mcr-1*- SB-R | CGCTCATAGCCATTGAAGCTG |  | This study |
